# Review of the Long-term Effects of Autologous Bone-Marrow Mononuclear Cell Implantation on Clinical Outcomes in Patients with Critical Limb Ischemia

**DOI:** 10.1038/s41598-019-44176-5

**Published:** 2019-05-22

**Authors:** Farina Mohamad Yusoff, Masato Kajikawa, Shogo Matsui, Haruki Hashimoto, Shinji Kishimoto, Tatsuya Maruhashi, Moniruddin Chowdhury, Kensuke Noma, Ayumu Nakashima, Yasuki Kihara, Taijiro Sueda, Yukihito Higashi

**Affiliations:** 1 Department of Cardiovascular Regeneration and Medicine, Research Institute for Radiation Biology and Medicine, Hiroshima University, Hiroshima, Japan; 2 Department of Cardiovascular Medicine, Hiroshima University Graduate School of Biomedical Sciences, Hiroshima, Japan; 3 Department of Medicine & Centre for Research on Non-Communicable Diseases, Faculty of Medicine & Health Sciences, Universiti Tunku Abdul Rahman, Selangor, Malaysia; 4 Division of Regeneration and Medicine, Medical Center for Translational and Clinical Research, Hiroshima University Hospital, Hiroshima, Japan; 5 Department of Surgery, Hiroshima University Graduate School of Biomedical Sciences, Hiroshima, Japan

**Keywords:** Cardiology, Risk factors

## Abstract

Critical limb ischemia (CLI) is associated with a high risk of limb amputation. It has been shown that cell therapy is safe and has beneficial effects on ischemic clinical symptoms in patients with CLI. The aim of this study was to further investigate the outcomes of intramuscular injection of autologous bone-marrow mononuclear cells (BM-MNCs) in a long-term follow-up period in atherosclerotic peripheral arterial disease (PAD) patients who have no optional therapy. This study was a retrospective and observational study that was carried out to evaluate long-term clinical outcomes in 42 lower limbs of 30 patients with atherosclerotic PAD who underwent BM-MNC implantation. The median follow-up period was 9.25 (range, 6–16) years. The overall amputation-free rates were 73.0% at 5 years after BM-MNC implantation and 70.4% at 10 years in patients with atherosclerotic PAD. The overall amputation-free rates at 5 years and at 10 years after implantation of BM-MNCs were significantly higher in atherosclerotic PAD patients than in internal controls and historical controls. There were no significant differences in amputation rates between the internal control group and historical control group. The rate of overall survival was not significantly different between the BM-MNC implantation group and the historical control group. Implantation of autologous BM-MNCs is feasible for a long-term follow-up period in patients with CLI who have no optional therapy.

## Introduction

Critical limb ischemia (CLI), the clinical syndrome of peripheral arterial disease (PAD), is characterized by rest pain with or without tissue loss due to inadequate blood perfusion to the affected extremities. Disruption of blood supply occurs due to the progression of PAD over a period of several weeks to months^1^. It was estimated that there were about 220 million people with PAD worldwide in 2010 and the disease burden has grown by almost a quarter over the past decade^2^. The incidence of CLI therefore has been increasing.

Guidelines for identifying severity and for appropriate treatments have been updated throughout the years. The decision for therapy is made on the basis of guidelines recommended by Trans-Atlantic Inter-Societal Consensus II (TASC II), which was established in 2007^3^. Currently available options include risk modification techniques, exercises, pain and ulcer managements, and revascularization interventions that are performed via endovascular or bypass surgical approaches^4,5^. Patients who are diagnosed with CLI are at risk of major amputation if they do not receive specific treatment. Mortality rates as high as 20% within 6 months from diagnosis and exceeding 50% at 5 years have been reported for patients with CLI, whereas 1‐year mortality rates in non-revascularizable patients, so‐called no‐option CLI (NO-CLI) patients, have been reported to range from 20% to 40%^6,7^.

The use of cell therapy in patients with CLI has been investigated for over 15 years. This therapeutic angiogenesis approach aims to offer a solution for no-option CLI patients. Cell therapy strategies include autologous, allogenic, and gene therapies and other modalities from various sources. In general, the effectiveness of cell therapies tends to differ from one center to another.

There has been scarce information on the long-term outcomes for over 10 years in atherosclerotic patients with CLI who underwent implantation of bone-marrow mononuclear cells (BM-MNCs) in order to reduce the amputation rate and to improve overall survival. Therefore, we evaluated the long-term effects of intramuscular injection of autologous BM-MNCs on clinical outcomes in atherosclerotic PAD patients who had no optional therapy.

## Results

### Patient characteristics

Table 1 shows baseline characteristics of patients in the BM-MNC implantation group and the historical control group. There were significant differences in baseline parameters, including smoking status and insulin use, between the two groups. Other parameters were similar in the two groups.

**Table 1:** Clinical Characteristics of the Subjects.

Variable	Historical control (n = 20)	BM-MNC implantation (n = 30)	P value
Age, yr	65.4 ± 10.2	67.2 ± 9.2	0.51
Gender, men/women	16/4	19/11	0.35
Body mass index, kg/m^2^	23.8 ± 4.1	22.0 ± 3.5	0.11
Rutherford category, n (%)			N.A.
3	0 (0)	1 (3)	
4	1 (5)	9 (30)	
5	10 (50)	15 (50)	
6	9 (45)	5 (17)	
Medical history, n (%)			
Hypertension	19 (95)	26 (87)	0.64
Dyslipidemia	10 (50)	18 (60)	0.57
Diabetes mellitus	19 (95)	25 (83)	0.38
Previous myocardial infarction	12 (60)	15 (50)	0.57
Previous stroke	7 (35)	8 (27)	0.55
Chronic kidney disease	13 (65)	14 (47)	0.25
Hemodialysis	9 (45)	8 (27)	0.23
Smoker (pre)	15 (75)	13 (43)	0.04
Medications, n(%)			
Anti-coagulant	6 (30)	11 (37)	0.76
Anti-platelets	16 (80)	24 (80)	1.00
ACE inhibitors	1 (5)	5 (17)	0.38
ARBs	7 (35)	12 (40)	0.77
Calcium-channel blockers	5 (25)	13 (43)	0.24
Statins	8 (40)	7 (23)	0.23
Sulfonylurea/metformin/other	9 (45)	11 (37)	0.57
Insulin	10 (50)	6 (20)	0.03

BM-MNC indicates bone-marrow mononuclear cell; ACE, Angiotensin-converting enzyme; ARB, Angiotensin II receptor blocker.

### Overall major amputation-free survival rate

**Figure 1:**
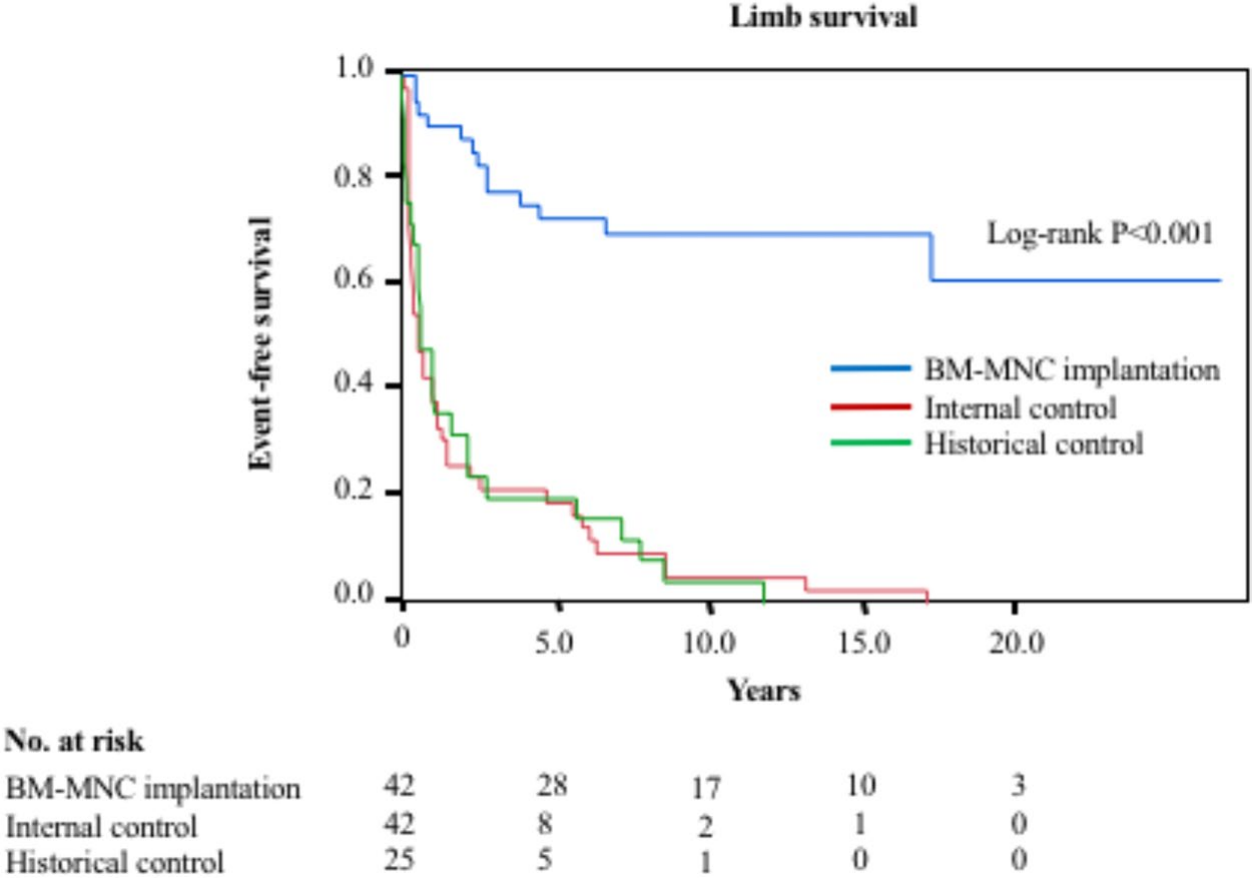
Major amputation free survival rates in atherosclerotic peripheral arterial patients who underwent bone-marrow mononuclear cell (BM-MNC) implantation, internal controls and historical controls.

The number of BM-MNCs implanted into ischemic limbs was 1.8 × 10^9^ ± 0.5 × 10^9^, and the number of CD34 cells was 3.5 × 10^7^ ± 1.4 × 10^7^. The median follow-up period was 9.25 (range, 6–16) years. Kaplan-Meier analysis showed that the major amputation-free survival rate was higher in atherosclerotic PAD patients who underwent BM-MNC implantation than in the internal controls and historical controls without cell therapy (Fig. 1). The overall major amputation-free survival rates were 73.0% at 5 years and 70.4% at 10 years in atherosclerotic PAD patients with BM-MNC implantation. The overall major amputation-free rates at 5 years and at 10 years were significantly higher in atherosclerotic PAD patients with BM-MNC implantation than in internal controls and historical controls. There were no significant differences in amputation rates between the internal control group and historical control group during the follow-up period. (Fig. 1).

### Overall survival rate

**Figure 2:**
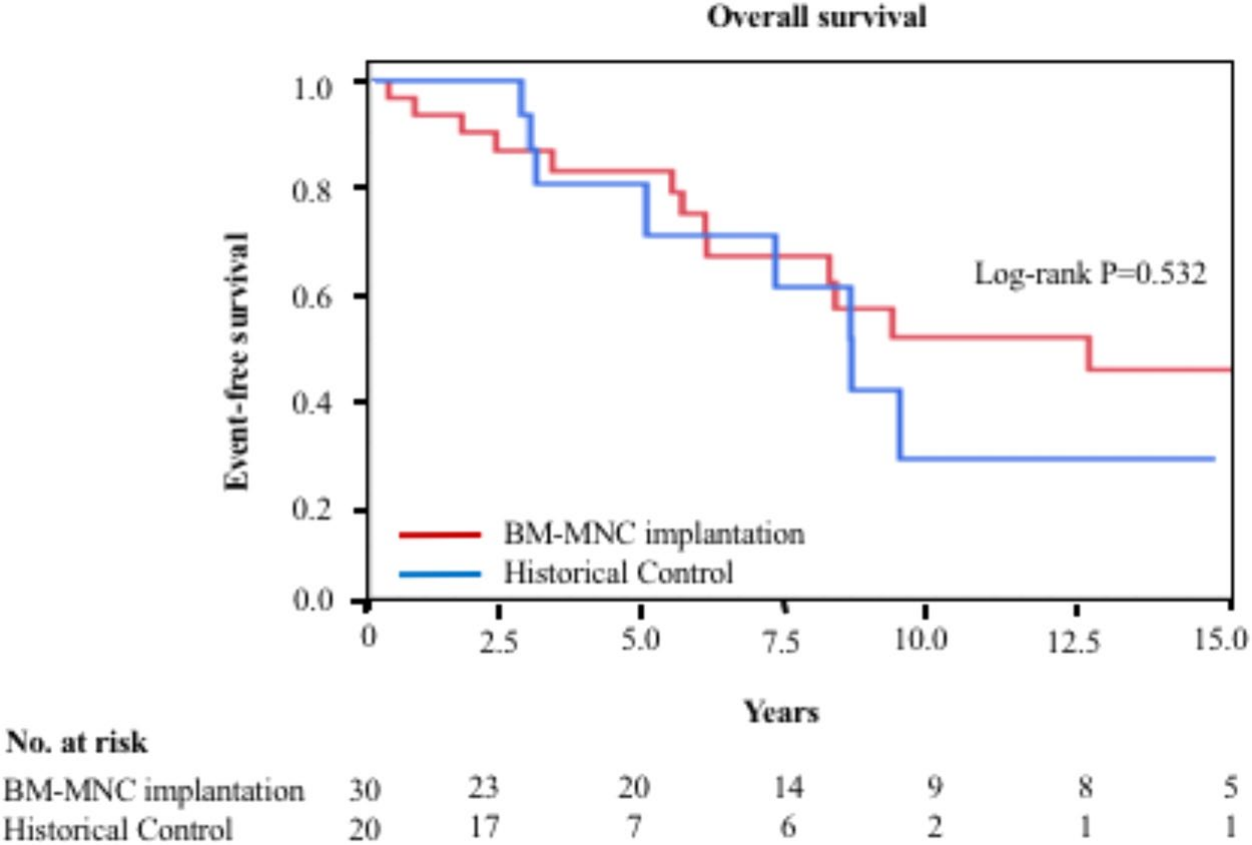
Overall survival rates in atherosclerotic peripheral arterial patients who underwent bone-marrow mononuclear cell (BM-MNC) implantation and historical controls.

The 30 patients with atherosclerotic PAD had a significantly higher long-term survival rate than that of the historical controls (Fig. 2). The 5-year overall survival rates were 82.5% in atherosclerotic PAD patients who underwent BM-MNC implantation and 70.0% in historical controls. The 10-year survival rate showed a decline in the atherosclerotic PAD patients who underwent BM-MNC implantation. Overall survival rates at 10 years were 50.2% in the BM-MNC implantation group and 26.7% in the historical control group. The causes of death throughout the years in atherosclerotic patients PAD with BM-MNC implantation (Table 2).

**Table 2:** Etiology of Deaths in Patients with BM-MNC Implantation and Historical Controls without BM-MNC Implantation over 10 Years.

Cause of death	Historical control (n = 8)	BM-MNC implantation group (n = 17)
Myocardial infarction, n	1	3
Heart failure, n	1	3
Stroke, n	0	3
Sepsis, n	2	4
Malignancy, n	0	1
Upper gastrointestinal bleed, n	0	1
Lower gastrointestinal bleed, n	0	1
Intestinal obstruction, n	0	1
Renal failure, n	1	0
Unknown, n	2	0

BM-MNC indicates bone-marrow mononuclear cell.

Major cardiovascular events include death from cardiovascular disease, myocardial infarction and stroke.

### Acute adverse events

There were no severe acute adverse events in patients who underwent BM-MNC implantation. A review of the PAD database and patients’ hospital records showed that adverse events that occurred with a few days included slight fever elevation in four of the BM-MNC implantation patients, progression of pain at cell implantation lesions in three the patients, and vertigo in one patient.

## Discussion

We demonstrated the safety and beneficial effects of BM-MNC implantation over a long-term follow-up period in patients with CLI. The rate of major amputation was decreased by BM-MNC implantation in atherosclerotic PAD patients who had no optional therapy compared with that in internal controls and that in historical controls. The rate of overall survival was not significantly different between atherosclerotic PAD patients with BM-MNC implantation and historical controls.

An estimated 10–40% of CLI patients with symptoms have no treatment options for various reasons. Within this group, those who have advanced limb ischemia with severe underlying medical illness are left untreated and are in need of palliative care^8^. Nevertheless, a larger portion of patients in this group who have non-critical underlying medical illnesses are treated conservatively in view of previously failed conventional therapies or therapies not attempted or are given the option of amputation. The cost of living with amputated limbs is higher, and amputation renders patients incapable of being productive individuals with an even higher risk of mortality^9,10^.

Cell therapy for CLI patients was first introduced in early 2000, and Tateishi-Yuyama *et al*. were the first to report the efficacy of intramuscular implantation of autologous BM-MNCs in patients with CLI in 2002^11^. Since then, many clinical research centers have adopted similar approaches with the hope of acquiring more evidence that cell therapy can be provided as an option for patients with NO-CLI. The Therapeutic Angiogenesis by Cell Transplantation (TACT) trial demonstrated that cell therapy using intramuscular implantation of BM-MNCs leads to extension of the amputation-free period and improvements in pain, ulcer size, and pain-free walking distance^11^. The safety and efficacy of BM-MNC implantation are not inferior to those of conventional revascularization therapies and are more efficient in patients with Buerger disease than in patients with atherosclerotic PAD^12^. Throughout the years, many centers have continued to investigate cell therapy for CLI. There have been mixed conclusions due to the variations in protocols for cell therapies in trials^13–18^.

The initial review by Idea *et al*. showed improvements in clinical symptoms of pain, transcutaneous oxygen pressure and early amputation-free rate, and survival outcomes were predominantly better than those in patients with Buerger disease^19^. Multivariate analyses also revealed less favorable outcomes in older patients, patients with diabetes mellitus and patients on hemodialysis. Previous reviews showed that atherosclerotic PAD patients with underlying severe medical conditions had less favorable outcomes.

In the present study, we followed up the general outcomes of patients who had undergone BM-MNC implantation and had received subsequent medical care with risk modifications over a period of more than 10 years. Long-term review revealed that patients who underwent cell therapy had significantly better limb survival than that of internal controls for limbs that were deemed unsalvageable and/or for amputation. Further analyses utilizing historical controls also showed a trend similar to that for the internal controls. However, cell therapy did not alter the survival rate of those patients.

Despite advancements in conventional interventions for patients who have options for treatment, there remain a large number of patients with CLI who need limb amputation. Indeed, patients with CLI have high rates of mortality and limb loss (almost one in five patients during a median follow-up period of 1 year)^6,7^. A few recent studies on utilization of cell therapy for patients with CLI have been carried out to provide solutions to NO-CLI^14–16^. Those studies suggest that the risk of major amputation was decreased by cell therapy in patients presenting with non-revascularizable CLI and that there is therapeutic potential in patients with type 2 diabetic who have CLI.

In the present study, we showed major amputation-free survival and overall survival rates in internal controls and PAD patients without BM-MNC implantation as historical controls. In the fields of therapy for leukemia and advanced cancers, internal controls are used to evaluate the efficacy of treatment when randomized control trials have not been performed. Interestingly, the rates of amputation were not significantly different between the internal control group and the historical control group during the follow-up period. These findings suggest that an internal control group is more useful than a historical control group for evaluating the effects of BM-MNC implantation on clinical outcomes in CLI patients who have no optional therapy.

BM-MNCs are a population of cells that include hematopoietic progenitor cells, lymphoid cells, monocytes, endothelial progenitor cells and cells of non-hematopoietic lineage. The progenitor cells are known to be multipotent and have effects in cellular interactions through autocrine and paracrine mechanisms. Bone marrow cells are recruited to the injured tissue in response to large amounts of cytokines and growth factors to prevent apoptosis, to provide cytoprotection of viable cells, to elicit anti-inflammatory effects and to reduce fibrosis^20^. It is thought that recruitment of specific stem cells leads to stimulation of angiogenesis. Shintani *et al*. showed that direct local implantation of autologous BM-MNCs augmented angiogenesis and collateral vessel formation in an ischemic limb model^21^. BM-MNCs have a paracrine effect on resident endothelial cells by secretion of angiogenic growth factors and cytokines to increase neo-vessel formation at the capillary level. These mechanisms lead to improvement of blood supply to the ischemic tissue. The ischemic tissue is unable to recruit the required cells for repair and for remaining viable. As the ischemic tissue begins to die, an inadequate cellular response leads to further deterioration of the affected limb. Implantation of BM-MNCs into the ischemic limbs promotes the acute phase of paracrine-mediated stimulation by the direct availability of a mononuclear cell population. Promotion of postnatal neovascularization by sheer stress and supply of angiogenic cytokines after BM-MNC implantation lead to an increase in collateral blood vessel formation, with utmost importance at the microcirculation level. These cells do not remain in the tissue in the long term. As the collaterals become mature and stable, blood supply to the affected tissue is improved. With the collateral blood vessels and adequate inflow of blood supply, the threatened limbs are no longer deemed critical for amputation. The availability of collaterals that are established after the acute phase of BM-MNC implantation would continue to assist the homeostasis in microcirculation of the tissue to remain viable. We speculate that avoidance of major amputation during the critical phase of the threatened limb along with optimized medical care enables avoidance of major amputation in the long term. There has been no specific biology study to address the mechanism of the long-term benefit. Further study is needed to determine the precise mechanism of the long-term benefit of BM-MNC implantation.

This study has a number of limitations. This study was a retrospective and observational study, not a prospective and randomized study. Although the number of study subjects was small, we clearly showed beneficial effects of BM-MNC implantation for the prevention of major amputation in patients with CLI who had no other treatment option. We performed BM-MNC implantation in patients with atherosclerotic PAD according to inclusion and exclusion criteria and a treatment strategy using an ideal protocol of the TACT trial^11^. However, we retrospectively corrected the data for historical controls with no inclusion and exclusion criteria. We cannot deny the possibility that favor or against limb amputation made was an uncertain decision in historical controls and that various factors influenced the treatment strategy or time to amputation in both patients with BM-MNC implantation and historical controls. Assessments of functional parameters (e.g., pain score, ulcer healing, and maximum walking distance) and perfusion indices (e.g., ankle-brachial index, transcutaneous oxygen pressure, and skin perfusion pressure) would enable more specific conclusions concerning the roles of BM-MNC implantation in the prevention of major amputation in patients with CLI to be drawn. Evaluation of cardiovascular outcomes and onset of cancer using a prospective, randomized, and controlled study design is needed. In addition, we were unfortunately not able to obtain more accurate information on comorbidities other than survival and major amputation during the long-term follow-up period. More information on comorbidities (e.g., incidence of cancer or autoimmune disease) would support the safety hypothesis for BM-MNC implantation.

## Conclusions

Intramuscular injection of BM-MNCs is safe and has beneficial effects on clinical symptoms for a long-term follow-up period in patients with CLI who have no optional therapy. Autologous implantation of BM-MNCs improves the rate of amputation-free survival in patients with no-option CLI. BM-MNC implantation in these patients tends to improve the amputation-free survival rate and survival rate, but results are not so good for patients with atherosclerotic PAD due to patients’ background of medical complications. The therapy has so far yielded neutral or at most marginally positive outcomes in patients with atherosclerotic PAD.

## Recommendations

Cell therapy has the potential to modify the natural history of intractable CLI. Besides the trend for improvements of local effects, the possibility of systemic effects of cell therapy such as effects on the cardiovascular system may contribute to improvements in underlying medical conditions. Procedures should be performed in highly skilled centers (adequately trained staff and a well-informed multidisciplinary team) to ascertain the safety and efficacy. There is a need to optimize patient indications, the cell dose regimen, and the delivery and route system, which are crucial for a positive outcome, with the aim of providing a solution to “NO-CLI”.

## Methods

### Study design

This study was a retrospective, observational and non-controlled study that was carried out to investigate the long-term clinical outcomes of BM-MNC implantation in atherosclerotic PAD patients with CLI. We previously showed the early safety and beneficial effects of BM-MNC implantation in PAD patients with CLI^19^. In this study, additional data for patients were obtained to evaluate the clinical outcomes in a long-term follow-up period. We assessed overall survival and major amputation-free survival in CLI patients who received cell therapy. We also investigated the causes of death in patients during a follow-up period more than 10 years. Further analysis of disease-specific amputation-free survival in patients with atherosclerotic PAD was performed to identify limb survival projections compared with those in internal controls who are same limbs (diagnosed for amputation) time analysis without BM-MNC implantation and those in historical controls without BM-MNC implantation.

### Study subjects

From May 2002 to April 2014, 30 patients with atherosclerotic PAD who had no option for revascularization underwent BM-MNC implantation. In the 30 patients, further statistical analyses were carried out for their 42 specific treated limbs. Furthermore, historical data for 20 patients with 25 affected limbs were retrospectively obtained up to August 2018 from the Hiroshima University Hospital PAD database. Atherosclerotic PAD was diagnosed on the basis of the guidelines of TASC II^3^. We ruled out vasculitis and hypercoagulable states. In all patients, angiography was performed to confirm limb ischemia. CLI was defined according to TASC II guidelines. We defined major amputation as above-the-ankle amputation. We performed BM-MNC implantation in patients with atherosclerotic PAD under inclusion and exclusion criteria and a treatment strategy using an ideal protocol of the TACT study^11^. Indication for BM-MNC implantation was decided by the Hiroshima Vascular Function Study members that included cardiologists, cardiovascular surgeons, plastic surgeons, dermatologists, radiologists and anesthesiologists. The ethics committee of Hiroshima University Graduate School of Biomedical Sciences approved the study protocol. Informed consent for participation in the study during the progress of the clinical trial was obtained from all subjects.

### Cell therapy: Autologous BM-MNC implantation

Isolation of BM-MNCs and implantation of BM-MNCs in CLI patients were performed as previously described^11,19^. All methods were performed in accordance with the relevant guidelines and regulations.

### Internal control

The internal control was an ischemic leg similar to that in which BM-MNC implantation was performed. The major amputation-free period was defined as the day on which major amputation was decided until the day BM-MNC implantation was performed.

### Historical control

Data for historical controls were obtained from Hiroshima University Hospital PAD database. Historical controls were patients with atherosclerotic PAD who had no optional therapy without BM-MNC implantation.

### Statistical analysis

Results are presented as frequency for categorical variables and means ± standard deviation. The chi-squared test or Fisher’s exact test was used to compare categorical variables. ANOVA was used for multiple groups and the t test was used to compare continuous variables in two groups. The Kaplan-Meier method was used for time-to-event endpoint analyses. We used a log-rank test to compare amputation-free survival and overall survival rates between the groups. Data were processed using JMP version 13.0 software (SAS Institute Cary, NC, USA). All statistical values were two-sided. A probability value of <0.05 was considered statistically significant.

## References

[1] Kinlay S (2016). Management of critical limb ischemia. Circ Cardiovasc Interv..

[2] Higashi Y (2017). Two-year follow-up of vascular events in peripheral arterial disease treated with antiplatelet agents: a prospective observational multicenter cohort study (SEASON). Scientific Reports.

[3] Norgren L (2007). Inter-society consensus for the management of peripheral arterial disease (TASC II). Eur J. Vasc Endovasc Surg..

[4] 2011 Writing Group Members, 2005 Writing Committee Members & ACCF/AHA Task Force (2011). 2011 ACCF/AHA focused update of the guideline for the management of patients with peripheral artery disease (updating the 2005 guideline): a report of the American College of Cardiology Foundation/American Heart Association Task Force on Practice Guidelines. Circulation.

[5] Adam DJ (2005). Bypass versus angioplasty in severe ischaemia of the leg (BASIL): multicentre, randomised controlled trial. The Lancet.

[6] Abu Dabrh AM (2015). The natural history of untreated severe or critical limb ischemia. J. Vasc Surg..

[7] Golomb BA, Dang TT, Criqui MH (2006). Peripheral arterial disease: morbidity and mortality implications. Circulation.

[8] Campbell WB, Verfaillie P, Ridler BM, Thompson JF (2000). Non-operative treatment of advanced limb ischaemia: the decision for palliative care. Eur J. Vasc Endovasc Surg..

[9] Mahoney EM (2008). One- year costs in patients with a history of or at risk for atherothrombosis in the United States. Circ Cardiovasc Qual Outcomes.

[10] Jordan RW, Marks A, Higman D (2012). The cost of major lower limb amputation: a 12-year experience. Prosthetics and Orthotics International.

[11] Tateishi-Yuyama E (2002). Therapeutic angiogenesis for patients with limb ischaemia by autologous transplantation of bone-marrow cells: a pilot study and a randomized controlled trial. The Lancet.

[12] Matoba S (2008). Long-term clinical outcome after intramuscular implantation of bone marrow mononuclear cells (Therapeutic Angiogenesis by Cell Transplantation [TACT] trial) in patients with chronic limb ischemia. Am Heart J..

[13] Fadini GP, Agostini C, Avogaro A (2010). Autologous stem cell therapy for peripheral arterial disease meta-analysis and systematic review of the literature. Atherosclerosis.

[14] Ozturk A (2012). Therapeutical potential of autologous peripheral blood mononuclear cell transplantation in patients with type 2 diabetic critical limb ischemia. J. Diabetes Complications.

[15] Samura M (2017). Therapeutic strategies for cell-based neovascularization in critical limb ischemia. J. Transl Med..

[16] Peeters Weem SM, Teraa M, de Borst GJ, Verhaar MC, Moll FL (2015). Bone marrow derived cell therapy in critical limb ischemia: A Meta-analysis of Randomized Placebo Controlled Trials. Eur J. Vasc Endovasc Surg..

[17] Pignon B (2017). Autologous bone marrow mononuclear cell implantation and its impact on the outcome of patients with critical limb ischemia: results of a randomized, double-blind, placebo-controlled trial. Circ J..

[18] Dong Z (2018). Purified CD34+ cells versus peripheral blood mononuclear cells in the treatment of angiitis-induced no-option critical limb ischaemia: 12-Month results of a prospective randomised single-blinded non-inferiority trial. EBioMedicine.

[19] Idei N (2011). Autologous bone-marrow mononuclear cell implantation reduces long-term major amputation risk in patients with critical limb ischemia: a comparison of atherosclerotic peripheral arterial disease and buerger disease. Circ Cardiovasc Interv..

[20] Takahashi M (2006). Cytokine produced by bone marrow cells can contribute to functional improvement of the infracted heart by protecting cardiomyocytes from ischemic injury. Am J Physiol Heart Circ Physiol..

[21] Shintani S (2001). Augmentation of postnatal neovascularization with autologous bone marrow implantation. Circulation.

